# Supplementary material for: Insights to the Structural Basis for the Stereospecificity of the Escherichia coli Phytase, AppA
Source: Int J Mol Sci. 2022 Jun 6;23(11):6346. doi: 10.3390/ijms23116346 (PMC9181005; doi:10.3390/ijms23116346)
Supplement: Supplementary file 1 [file ijms-23-06346-s001.zip › ijms-1744367-supplementary-EDITED-220606.pdf]

SUPPLEMENTARY INFORMATION

Insights to the structural basis for the stereospecificity of the *Escherichia coli* phytase, AppA

Isabella M. Acquistapace[1], Emma J. Thompson[1], Imke Kühn[2], Mike R. Bedford[3], Charles A. Brearley[1] & Andrew M. Hemmings[1,4,5,*]

[1] *School of Biological Sciences, University of East Anglia, Norwich NR4 7TJ. U.K.*

[2] *AB Vista, Feldbergstrasse, 64293 Darmstadt, Germany.*

[3] *AB Vista, Blenheim Road, Marlborough SN8 4AN, U.K.*

[4] *School of Chemistry, University of East Anglia, Norwich NR4 7TJ. U.K.*

[5] *College of Food Science and Technology, Shanghai Ocean University, Shanghai 201306, China*

**Correspondence: a.hemmings@uea.ac.uk*

SUPPLEMENTARY TABLES

Table S1. X-ray Data Collection and Structure Refinement Statistics

**Mutant**	**WT**	**WT**	**D304A**	**D304A**	**D304A T305E**	**D304E**	**T305E**
**Variant**	**WT-Pi**	**WT-IHS**	**HAT-NEP**	**HAT-IHS**	**HAE-IHS**	**HET-HIS**	**HDE-IHS**
PDB entry code	7Z1J	7Z2S	7Z32	7Z2T	7Z2W	7Z3V	7Z2Y
Beamline	I04	I03	I03	I03	I03	I03	I04
Wavelength	0.9795	0.9762	0.9763	0.9762	0.9762	0.9762	0.9795
Resolution range	38.3 - 1.85 (1.92 - 1.85)	38.4 - 1.72 (1.78 - 1.72)	38.5 - 1.85 (1.92 - 1.85)	41.1 - 1.41 (1.46 - 1.41)	38.2 - 1.42 (1.47 - 1.42)	65.3 - 2.60 (2.69 - 2.60)	38.2 - 1.86 (1.93 - 1.86)
Space group	P 1 21 1	P 1 21 1	P 1 21 1	P 1 21 1	P 1 21 1	P 1 21 1	P 1 21 1
Unit cell	63.6 47.6 65.7 90 101.0 90	63.7 47.8 65.6 90 100.8 90	63.8 48.0 66.0 90 101.1 90	63.5 47.4 65.6 90 100.8 90	63.6 47.5 65.5 90 100.5 90	63.7 44.4 66.6 90 101.3 90	63.6 47.5 65.4 90 100.7 90
Total reflections	232047 (23025)	266419 (23781)	103436 (10454)	215532 (20898)	231879 (23026)	75247 (7474)	94958 (9599)
Unique reflections	33226 (3295)	40902 (3952)	32709 (3234)	73471 (7274)	71314 (7086)	11458 (1117)	32339 (3215)
Multiplicity	7.0 (7.0)	6.5 (6.0)	3.2 (3.2)	2.9 (2.9)	3.3 (3.2)	6.6 (6.7)	2.9 (3.0)
Completeness / %	99.96 (99.97)	98.91 (97.12)	97.12 (96.74)	99.17 (98.89)	98.07 (98.29)	99.78 (99.46)	98.22 (96.52)
Mean I/sigma(I)	7.84 (1.77)	10.04 (1.65)	8.05 (2.12)	10.85 (1.83)	9.99 (1.42)	6.04 (3.51)	5.55 (1.57)

Wilson B-factor	15.48	20.73	16.31	13.08	14.96	15.61	12.40
R-merge	0.2263 (1.07)	0.1324 (1.123)	0.1327 (0.6246)	0.06784 (0.724)	0.06782 (0.8522)	0.4109 (1.689)	0.142 (0.7293)
R-meas	0.2446 (1.156)	0.1442 (1.233)	0.1598 (0.7482)	0.08297 (0.8903)	0.08139 (1.024)	0.4464 (1.833)	0.1732 (0.8854)
R-pim	0.09171 (0.4328)	0.05646 (0.5014)	0.08789 (0.4071)	0.04709 (0.5106)	0.04443 (0.5608)	0.1724 (0.7037)	0.09798 (0.4965)
CC1/2	0.988 (0.573)	0.996 (0.517)	0.991 (0.59)	0.997 (0.494)	0.998 (0.488)	0.958 (0.511)	0.987 (0.567)
CC*	0.997 (0.853)	0.999 (0.826)	0.998 (0.862)	0.999 (0.813)	1.000 (0.81)	0.989 (0.822)	0.997 (0.851)
Reflections used in refinement	33225 (3295)	40901 (3952)	32706 (3234)	73470 (7274)	71314 (7086)	11434 (1111)	31926 (3132)
Reflections used for R-free	1628 (175)	1967 (171)	1676 (173)	3692 (356)	3574 (355)	578 (57)	1567 (170)
R-work	0.1480 (0.1900)	0.1504 (0.2453)	0.1631 (0.2353)	0.1477 (0.2374)	0.1522 (0.2437)	0.1937 (0.2341)	0.1831 (0.2710)
R-free	0.2268 (0.2958)	0.2066 (0.3494)	0.2244 (0.3109)	0.1803 (0.2779)	0.1871 (0.2787)	0.2733 (0.3722)	0.2572 (0.3651)
CC(work)	0.965 (0.882)	0.969 (0.850)	0.964 (0.840)	0.971 (0.845)	0.967 (0.844)	0.939 (0.839)	0.948 (0.808)
CC(free)	0.952 (0.792)	0.959 (0.576)	0.934 (0.761)	0.969 (0.781)	0.966 (0.751)	0.854 (0.764)	0.934 (0.747)

Number of non-hydrogen atoms	3672	3524	3555	3665	3567	3304	3682
macromolecules	3112	3101	3155	3140	3148	3095	3113
ligands	13	38	1	80	80	115	79
solvent	547	385	399	466	360	100	496
Protein residues	406	405	412	406	406	404	405
RMS(bonds)	0.007	0.007	0.007	0.005	0.006	0.008	0.009
RMS(angles)	0.9	0.85	0.87	0.85	0.92	1.08	1.12
Ramachandran favored (%)	98.51	98.75	98.77	98.51	99.01	94	97.77
Ramachandran allowed (%)	0.99	1.25	1.23	1.24	0.74	4.25	1.74
Ramachandran outliers / %	0.5	0	0	0.25	0.25	1.75	0.5
Rotamer outliers / %	0.88	1.18	0.58	0.29	0.57	4.14	0.88
Clashscore	4.01	4.16	5.4	5.64	5.31	21.96	9.2
Average B-factor	19.83	25.8	21.82	18.78	19.77	14.63	16.88
macromolecules	17.76	24.06	20.38	16.47	17.92	14.41	14.45

ligands	25.52	48.74	23.09	39.68	37.98	22.13	33.7
solvent	31.5	37.53	33.18	31.65	32.97	13.13	29.64
TLS groups	0	0	0	0	0	2	0

Table S2. Composition of specificity pockets in the active site of AppA. Residues within 5 Å of the sulfate groups of $InsS_6$ in the AppA-HDT:$InsS_6$ complex. Note this is a slightly more conservative distance cutoff criterion than used in preparation of figure 2.

Specificity pocket	#	Pocket residues								
A	7	H17	R16	R20	R92	H303	D304	T305		
B	9	R16	R20	S215	M216	E219	H250	Q253	F254	D304
C	5	T23	K24	R20	S215	M216				
D	2	T23	K24							
E	3	T23	K24	R92						
F	5	D88	D90	R92	H303	T305				

SUPPLEMENTARY FIGURES

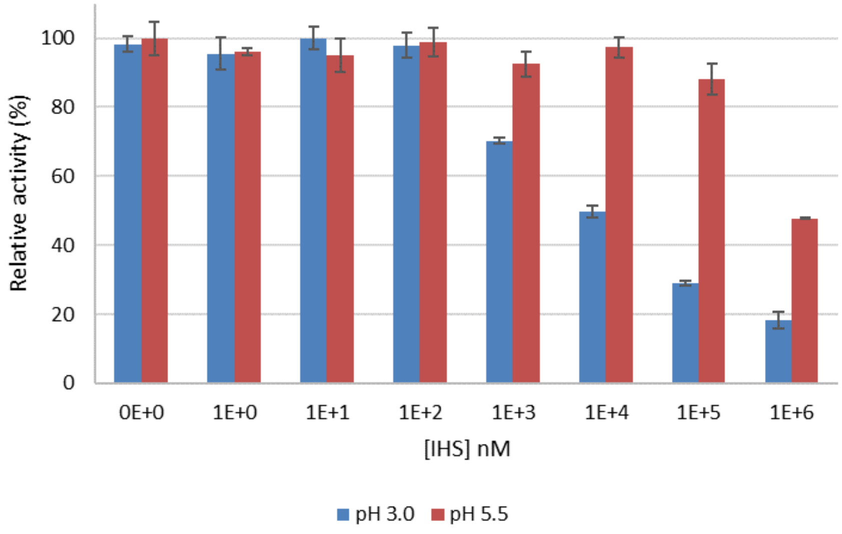

Figure S1. Inhibition of AppA by *myo*-inositol hexasulfate. Relative activity (%) is plotted as a function of inhibitor concentration (nM). Blue bars show relative activity measured in glycine buffer pH 3.0 while red bars show activity in sodium acetate buffer pH 5.5. Error bars represent standard deviations of triplicate measurements.

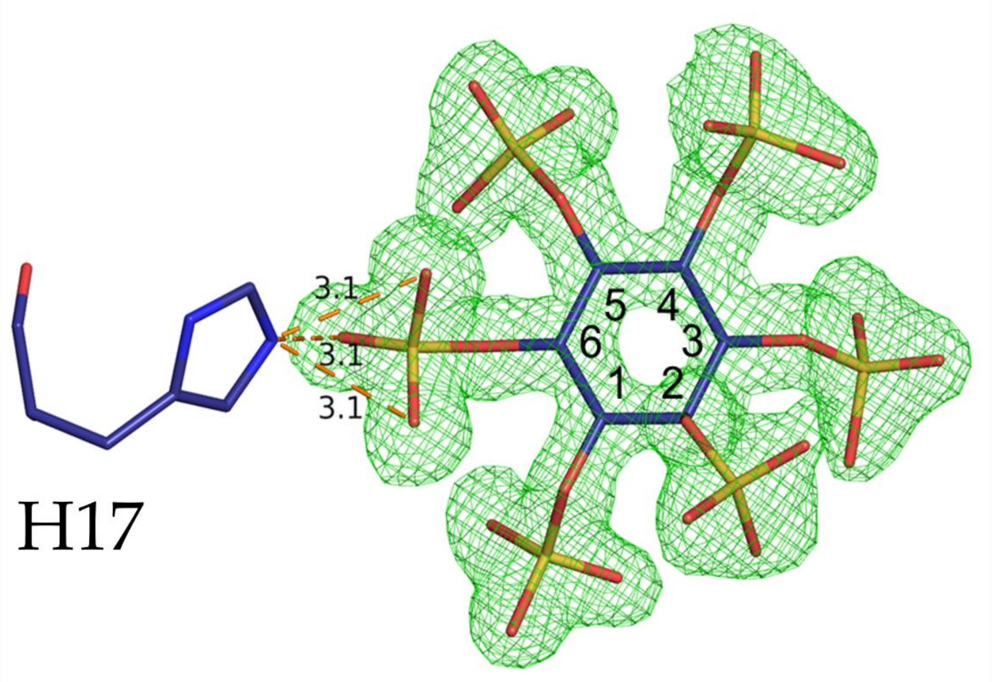

Figure S2. Polder ligand omit map of the substrate analogue $InsS_6$ in complex with AppA. Fo-Fc omit map (green hatching) of the density surrounding $InsS_6$ shown at a contour level of 3.0 σ. The carbon atoms of the *myo*-inositol ring are numbered. The catalytic histidine H17 contacts the 1D-6-sulfate group with interaction distances (in Å) shown as dashed lines.

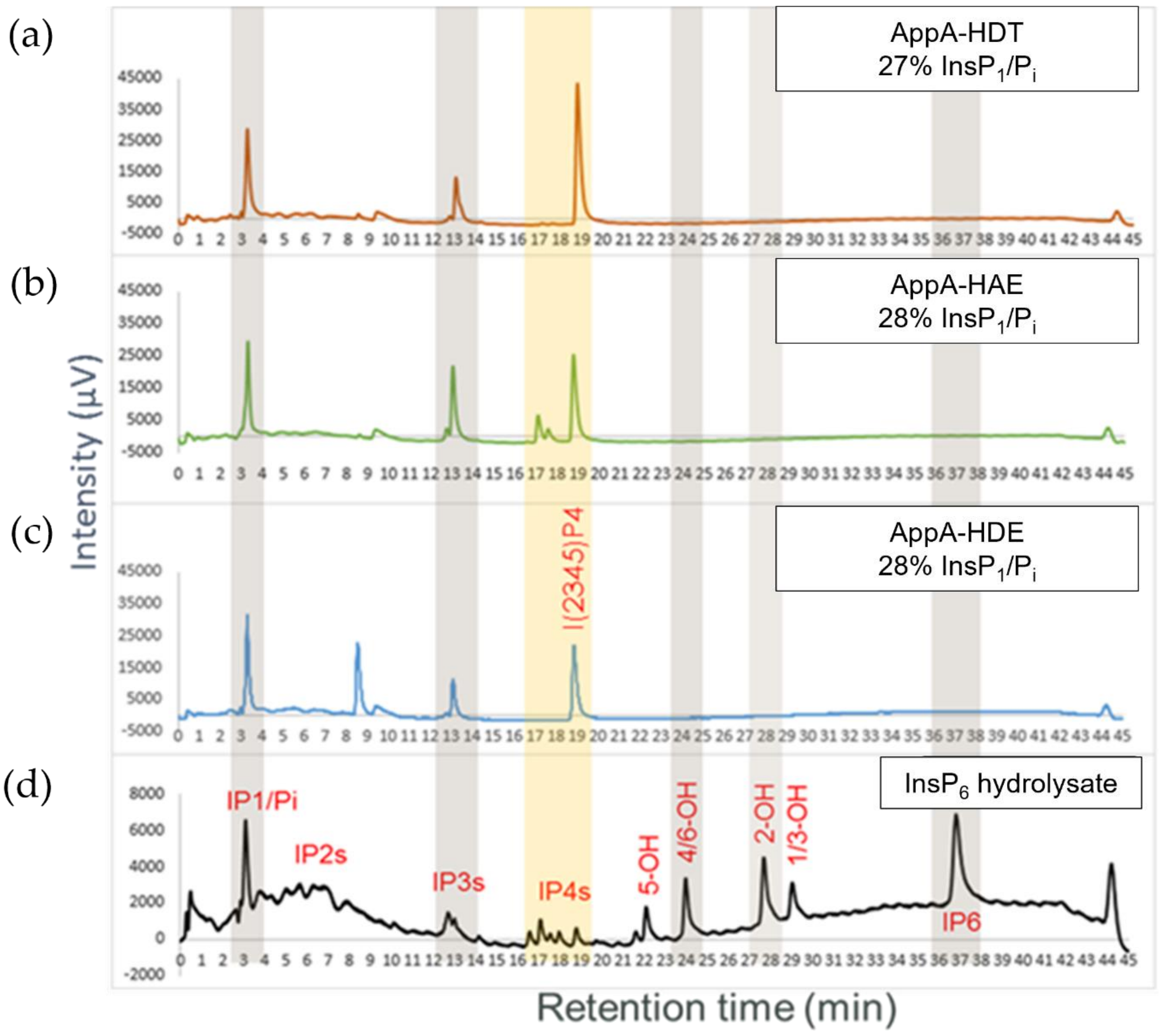

Figure S3. HPLC chromatograms of the products of extended hydrolysis of $InsP_6$ by wild type AppA and selected proton donor motif variants. Reactions stopped when total $InsP_1$/orthophosphate (labelled IP1/P_i) peak area is equal to 28 % of the total. **(a)** AppA-HDT (wild type) **(b)** AppA-HAE **(c)** AppA-HDE and **(d)** chromatogram of an acid hydrolysate of the substrate ($InsP_x$ standards) is shown for reference. The enzyme proton donor motif and the predominant $InsP_5$ peak area (%) are reported on the top right corner of each chromatogram. The elution volume ranges for the various inositol polyphosphates are highlighted by vertical coloured backgrounds (note that the notation for the presumed $InsP_5$ product is based on the identity of the free hydroxyl group of the intermediate). The nomenclature used to identify inositol polyphosphate intermediates in panels (c) and (d) is simplified for the purposes of clarity.

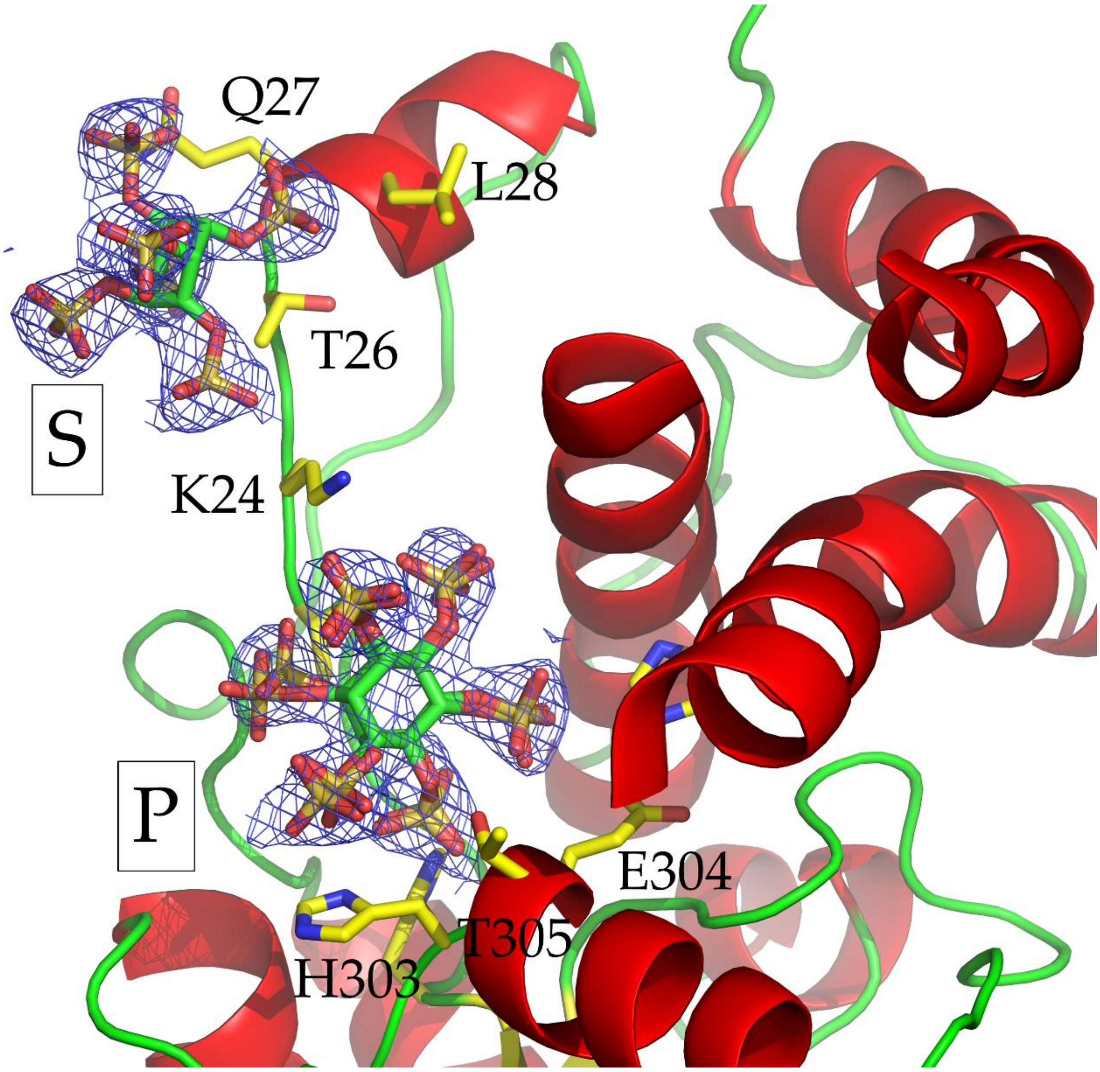

Figure S4. Conformational disorder in binding InsS6 inhibitor to the AppA-HET variant. The enzyme is shown in cartoon representation with β-strands, α-helices and random coil coloured yellow, red and green, respectively. The inhibitor molecules are shown in stick format with carbon atoms coloured yellow for the two copies of the inhibitor molecule in alternate conformations in the primary binding site (labelled P) and coloured magenta for the inhibitor in a secondary site (labelled S). The $2mF_o$-DF_c difference electron density map in the region of the bound inhibitor molecules is shown as a blue mesh contoured at 1.0 σ. Sidechains of residues in the proton donor motif and those in the vicinity of the secondary binding site are shown and labelled.

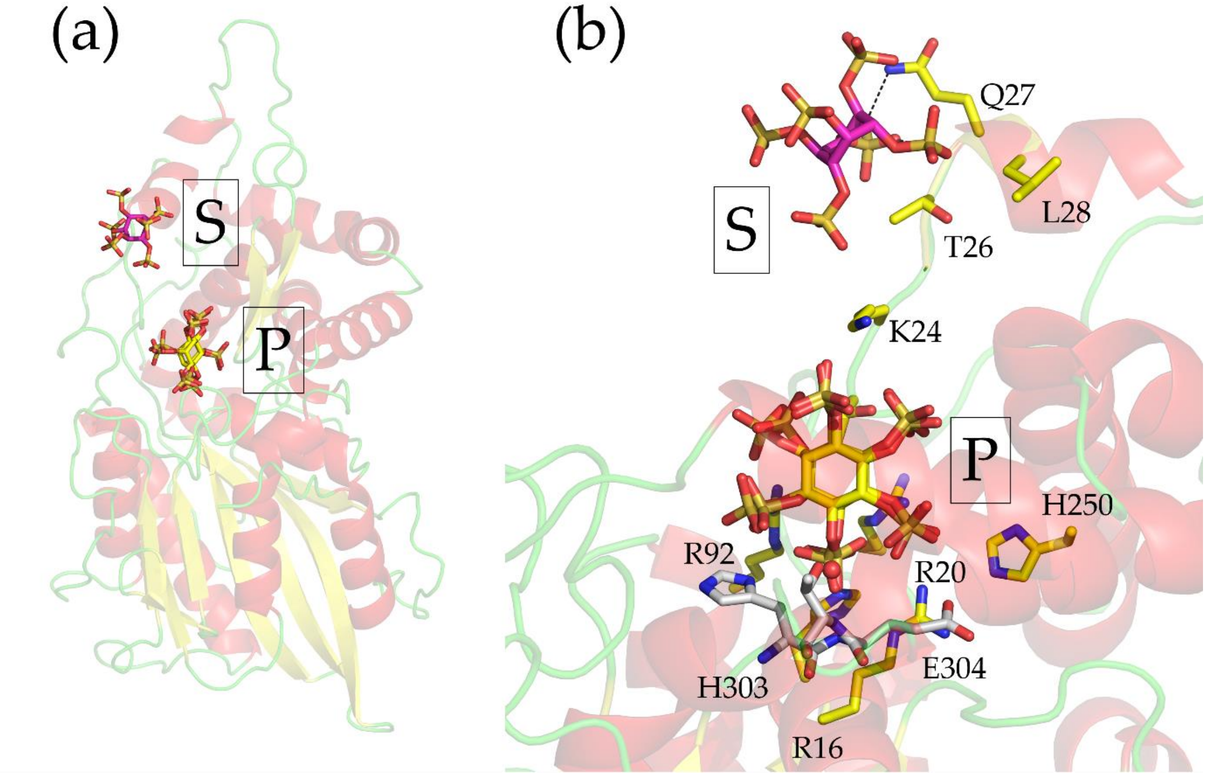

Figure S5. A secondary binding site for $InsS_6$ in AppA. (a) An overview of the structure of the complex of $InsS_6$ with the AppA-HET variant. The enzyme is shown in cartoon representation with β-strands,α-helices and random coil coloured yellow, red and green, respectively. The inhibitor molecules are shown in stick format with carbon atoms coloured yellow for inhibitors in the primary binding site (labelled P) and coloured magenta for the inhibitor in the secondary site (labelled S). **(b)** A close up of the binding sites. Colouring as in panel (a). Residues interacting with the ligands are labelled.
